# Impact of Bone Marrow Natural Killer Cells (NK); Soluble TNF-α and IL-32 Levels in Myelodysplastic Syndrome Patients

**DOI:** 10.31557/APJCP.2020.21.10.2949

**Published:** 2020-10

**Authors:** Salah Aref, Nada Khaled, Abdel Hady Al Gilany, Mohamed Ayed, Tarek Abouzeid, Doaa Attia

**Affiliations:** 1 *Hematology Unit, Clinical Pathology Department, Faculty of Medicine, Mansoura University, Egypt. *; 2 *Public Health and Biostatistics Department, Mansoura University, Egypt. *; 3 *Hematology Unit, Mansoura University Oncology Center, Mansoura University, Egypt. *

**Keywords:** NK cells, MDS, sTNF-α, sIL32, AML

## Abstract

**Background:**

Myelodysplastic syndromes (MDS) are complex clonal hemopoietic progenitor cell disorders that result from the evolution of aberrant clones which lead to leukemia. Disorders of the immune system serve important functions in the pathophysiology and progression of this disorder. This study aimed to assess the bone marrow natural killer cells percentage as well as soluble TNF-α and sIL-32 concentration levels in MDS patients.

**Methods:**

Bone marrow samples were obtained from 34 MDS; 12 MDS-AML and 10 controls. The percentage of total NK cells and mature NK cells were determined by flowcytometry. Bone Marrow soluble TNF-α and sIL-32 concentration levels were measured by ELISA.

**Results:**

The percentage of total NK and mature NK cells were significantly lower in MDS patients as compared to controls (P<0.001). The NK cells percentages were significantly related to MDS severity scores being lowest in high score followed by intermediate score and then low score (P<0.001). Moreover; the bone marrow sTNF-α and sIL-32 levels were higher in AML-MDS group; followed by MDS group then the control group and the differences are statistically significant (P<0.001 for both).

**Conclusion:**

The reduction in NK cells might have a role in AML evolution on the top of MDS. Likewise, the bone marrow sTNF-α; and sIL32 might have a role in MDS cytopenia.

## Introduction

Myelodysplastic syndrome (MDS) is a complex clonal hemopoietic progenitor cell disorder that result from the evolution of aberrant clones and is characterized by an ineffective hematopoiesis and frequent cell apoptosis in the bone marrow (BM) and manifested by blood cytopenia’s (Warlick and Miller, 2011). Chronic activation and innate immune signaling in hematopoietic cells is widely described as a risk of developing MDS. Recent reports with an evidence based support stated that there is direct role of inflammation in the pathogenesis of MDS (Barreyro et al., 2018). Furthermore; immunological derangement has been postulated as one of the causes of MDS transformation to acute leukemia (Lambert et al., 2016; Epling-Burnette et al 2007).

The immune defense from the human body to malignant cells is formed of two arms; the first is innate immunity and the second is adaptive immune response. Important attention for NK cells. Based on its ability to kill the malignant cells as well as due to its ability for production of proinflammatory cytokines (Fehniger et al., 2016). Natural Killer (NK) cells are central cells in both innate and adaptive response; this is due to its role in body defense against malignant transformation based on its potent antileukemic cytotoxic effectors. Killing of target cells by NK cells are independent on a HLA way (Kiladjian et al., 2006). A defect in NK cytotoxicity has been postulated in certain hematopoietic malignancies including multiple myeloma; acute myeloid leukemia and MDS (Tsirigotis et al., 2017; Bjorklund et al 2018; Carlsten and Järås , 2019). This defect is at least partially linked to a decreased or absent expression of some activating NK cells molecules, more particularly the so-called natural cytotoxicity receptors. based on the relative expression of CD16 and CD56; the human NK cells could be subdivided into different populations (Poli et al., 2009) which includes two subtypes: CD56dimCD16+ (localized mostly in peripheral blood, BM, and spleen, and principally involve a cytokines mediated response) express high killer immunoglobulin like receptors (KIR) being responsible for most NK cytotoxicity because of its high expression of KIR receptor and CD56^bright^ CD16^dim/negative^ (localized mostly in peripheral tissue sites and involved primarily in direct target cell cytolytic activity). NK cells recognize virally infected and neoplastic cells predominantly in a major histocompatibility (MHC)-unrestricted fashion by recognizing target cells having down-regulated MHC class I molecules (Aggarwal et al., 2016).

Several studies pointed out for the interrelation between soluble interleukin (sIL)-32; sTNF-α; NK cells percentage and cytopenia’s in MDS patients (Marcondes et al., 2008; Fehniger et al., 2016); however, no previous study assessed NK cells and NK subsets in parallel with TNF-α and IL-32 in the BM of AML-MDS patients. Therefore; the aim of this study was to assess the percentage of NK cells and mature NK cells as well as TNF-α and IL32 in the BM of MDS and AML-MDS patient group and normal BM controls. 

## Materials and Methods

This is a case control study; in which BM samples were obtained from 46 MDS patients (26 males and 20 females) with mean age 59.9±6.2 years and 10 controls (Refereed for hip replacement) (6 males; 4 females; mean age 58±7.2 years). The patients were followed up clinically for up to 36 months. Fourteen MDS patients transformed to AML (MDS-AML subgroup) and the remaining MDS subgroup (n=32) remains in the MDS state.

For the BM samples; the percentage of total NK cells and its subset mature NK cells were determined by flowcytometry. The BM sTNF-α and sIL-32 levels were determined by ELISA. 

The study was conducted at Mansoura University Oncology Center (MUOC), between January 2015 and January 2019 after taken informed consent. Exclusion criteria included patients with a history of autoimmune disease, HIV or solid organ transplantation or patients previously treated with chemotherapeutic agents.

The Revised International Prognostic Scoring System (IPPS-R) for MDS was used for stratification into prognostic subgroups (Greenberg et al., 2012). 

*Flow Cytometric Immunophenotypic Studies*

BM samples were collected on EDTA tubes from MDS; AML-MDS and controls. The analysis was done in the same day of sample collection. The analysis of BM samples was done for the percentage of T cells to total lymphocytes and NK cells to T cells and percentage of mature NK to total NK cells. Briefly; 5 μl of selected proper monoclonal antibodies to each tube containing 100 μl BM, incubated 20-30 minutes in dark at room temperature, washed with phosphate buffered saline (PBS) three times, and then added 300 μl phosphate buffered saline (PBS) before analysis. Isotopic control using IgG1 and IgG2 on each sample was done to exclude auto fluorescence.

The following tube (NK/T cell tube) was used; in which the following monoclonal antibodies were added (CD16/57 FITC, CD7 PE, CD4 PerCP-Cy5.5, CD2 PE-Cy7, CD56 APC, CD3 APC-H7, CD5 V450, CD8 V500; (BD Biosciences, San Jose, CA. Data were acquired using BD FACS Canto II flow cytometer (BD Bioscience, San Jose, CA), and 50,000 events were collected in most cases wherever possible. The data analysis was done by software obtained from BD FACSDiva (BD Bioscience, San Jose, CA). The gating strategy for lymphocytes were done based on CD45 expression and side scatter. The identification of T-cells was based on the finding of CD2 positive, CD3 positive, and NK cell subsets were identified by gating specifically on the CD7+, CD3− cells and evaluating expression of CD56 and CD16/57(mature NK cells: CD56dim CD16/57positive).

*Cytokines assays*

Cytokines in the BM plasma were obtained from studied patients’ groups before start of therapy and also from controls and then frozen at -80o till the time of analysis. After thawing, plasma concentration of sTNF-α and sIL-32 levels was measured using commercially available enzyme linked immunosorbent assay (ELISA) assay kits Quantikine (R&D Systems, Minneapolis, MN, USA) according to the manufacturer’s instructions. 

*Statistical Analysis*

Statistical analysis was performed using Stata 12 software (Stata Corp LP, College Station, TX). Comparisons of the median percentage of NK cells, and NK cell subsets; sTNF-α and sIL32 concentration levels were performed using Mann Whitney-U and Kruskal–Wallis tests. The correlation analyses were done by Spearman correlation.

## Results

*Clinicopathologic characteristics of the studied Patients*

The mean age of the MDS cohort was 59.5±6.4 years; they were 26 males and 20 females. Based on the revised MDS score the MDS group of patients was classified into 24 low risk MDS, 16 intermediate risk MDS, and 6 high risk MDS. Ten controls which include 6 males and 4 females; the mean age was 58±7.2 years.

*sTNF-α and sIL-32 levels BM levels*

The bone marrow concentration levels of sTNF-α and sIL-32 were significantly higher in the total MDS patients’ group as compared to controls (P<0.001). Moreover; the sTNF-α and sIL-32 BM levels were significantly related to MDS severity scores being the highest in MDS subgroup with high score followed by intermediate score and then low score and the differences was statistically significant (P< 0.001). On the other hand; the levels of sTNF-α and sIL-32 in the bone marrow did not significantly different between MDS subgroups and MDS-AML subgroup. 

*Percentages of total NK cells and mature NK*

The percentage of total NK cells was significantly lower in the total MDS patients’ group as compared to control group (P<0.001). On the other hand, the mature NK cells percentage was not significantly different in patient’s group and controls (P>0.05). In MDS-AML patient group the total NK cells percentage and mature NK cell percentage are significantly reduced as compared to MDS patient group (P<0.001).

*Correlation studies between bone marrow sTNF-α; sIL-32 levels and NK cells*

In MDS patients’ group sTNF-α and sIL-32 and total NK cells are negatively correlated to the blood absolute neutrophil counts ( r= - 44; -53; -43 respectively). While; in MDS group that transformed to AML group, only NK cells are negatively correlated to BM blast cells. (Table 4). 

**Table 1:** Comparison between Control and Patients’ Group

Parameter	Control (n=10) Mean ± SD	Total patients’ group (n=46) Mean ± SD	*P*-value
Sex n. (%)			
Female	4 (40.0%)	20 (43.5%)	P=0.5
Male	6 (60.0%)	26 (56.5%)	
Age (years)	58±7.2	59.9±6.2	P>0.05
TNF- α (ng/ml)	4.9±1.4	10.1±2.1	P≤0.001
IL-32 ng/ml	4.3±0.5	7.7±2.7	P≤0.001
Total lymphocytes count/cmm			
Median	1705	510	
(min-max)/cmm	(1250.0-2800.0)	(66.0- 653.0)	P≤0.001
T cells (Percentage of total lymphocytes)	66.95±13.4	79.1±7.1	P≤0.001
NK (Percentage of total lymphocytes)	15.4±4.1	8.99±4.8	P≤0.001
Mature NK (Percentage of total NK)	78.7±9.5	81.1±9.7	P=0.4

Comparison between groups was done by Mann-Whitney test

**Table 2:** Demographic and Laboratory Data in MDS Subgroups and AML-MDS Group

Parameter	Group I (n=24)	Group II (n=16)	Group III (n=6)	P value
	Mean ± SD	Mean ± SD	Mean ± SD	
Sex (N & %):				
Female	10 (41.7%)	7 (43.8%)	3 (50%)	P=0.1
Male	14 (58.3%)	9 (56.2%)	3 (50%)	
Age	57.8±7.2	60.5±5.8	62.7±3.6	P=0.1
TNF alpha(ng/ml)	8.7±0.8	10.5±1.5	12.0±0.8	P≤0.001
IL32 (ng/ml)	5.5±1.7	8.96±1.7	11.1±1.9	P≤0.001
Total lymphocytes count ×10^3^/cmm	574.4±178.5	451.9±99.6	398.3±122.9	P≤0.001
T lymphocytes (Percentage of total lymphocytes)	82.1±7.1	80.1±4.97^C^	74.7±4.8	P≤0.001
NK (Percentage of total lymphocytes)	11.8±5.1	8.7±2.3	5.8±3.4^A^	P≤0.001
Mature NK (Percentage of total NK)	83.7±6.1	84.6±3.7	85.7±1.7^C^	P>0.05

MET, Monte Carlo exact test; A, B, C, significant difference between the corresponding group by Bonferroni's post hoc multiple comparison

**Table 3:** Comparison between MDS and MDS that Transformed to AML

Parameter	MDS (n=32)	AML-MDS (n=14)	P value
	Mean ± SD	Mean ± SD	
Sex (N & %)			
Female	14 (43.8%)	6 (42.9%)	P=0.1
Male	18 (56.2%)	8 (57.1%)	
Age (years)	59.5±6.4	58.9±5.2	P=0.2
TNF alpha (ng/ml)	9.8±1.9	12.6±0.8	P≤0.001
IL32 (ng/ml)	7.4±2.6	10.1±1.4	P=0.01
T lymphocytes (Percentage of total lymphocytes)	80.4±6.5	70.6±3.7	P≤0.001
NK (Percentage of total lymphocytes)	9.9±4.6	3.0±2.1	P≤0.001
Mature NK (Percentage of total NK)	84.3±4.9	59.9±5.9	P≤0.001

**Table 4.** Correlation between Blood Absolute Neutrophil Counts in MDS Group as Well as Bone Marrow Blast Cells Counts in Secondary AML Groups and sTNF-α, sIL-32, NK % and Mature NK Cell Percentage

Parameter	Bone Marrow sTNF-α	Bone Marrow sIL-32	Bone Marrow total NK cells%	Bone Marrow Mature NK cells %
AML-MDS (n=14)				
Bone marrow blast cells percentage	R= 0.08	R= 0.013	R= - 0.53*	R= 0.15
MDS (n=32)				
Absolute blood neutrophil counts	R= - 0.44*	R= - 0.53*	R= - 0.43*	R= 0.11

Significant*

## Discussion

Many previous reports stated the role of immunity in evolution and progression of MDS into acute leukemia. Based on the reports that NK cells are able to kill cancer cells (Sanchez-Correa et al., 2016) and secrete proinflammatory cytokines as well as the recent discovery of NK cell memory which support the concept that the anti-cancer potential of NK cells can be enhanced long term (Fehniger et al., 2016; Romee et al., 2016). These findings attract our attention to test the hypothesis that NK cells might have a role in transformation of MDS to AML-MDS. 

 In the current study the mean percentage of total NK cells (Relative to total lymphocytes) and matures NK cells (relative to total NK cells) in the control BM was similar to that reported by Valiathan et al., (2014). However; it was lower than that reported by Aggrawal et al., (2016). This could be explained on the basis that the control BM samples in the previous study were obtained from NHL cases that were referred for BM staging; while in our study it was collected from normal BM refereed for hip replacement.

The percentage of total NK cells (relative to the total lymphocytes) in the BM samples were significantly higher in the controls as compared with that detected in the MDS or MDS subgroup that is transformed to AML. These findings were in agreement with that reported by Bourgeois et al., (2006) and Gleasor et al., (2014). However, these findings did not parallel with that reported by Aggrawal et al., (2016); who found insignificant differences between MDS patients and controls as well as in between the MDS subgroups. Previous study based on in vitro observations showed that alloreactivity NK cells killed effectively malignant cells derived from AML; multiple myeloma; chronic myeloid leukemia and chronic lymphocytic leukemia (Sanchez-Correa et al., 2016). On the other hand, self NK cells were proved to be functionally incompetent against most malignant cells; this was attributed to suppression by self-major histocompatibility molecules on the malignant cells. Furthermore; in certain myeloid malignancies some NK cells express the same cytogenetic abnormality and there for it was suggested to be originated from leukemic progenitor cells (Poli et al., 2009).

In the present study the NK cells percentages were significantly related to MDS severity scores; being the lowest in high score followed by intermediate score and then low score. These findings were different from that reported by Aggrawal et al., (2016) who stated there is no significant different between MDS categories or AML patients. This could be attributed to the differences in the pathophysiology of MDS cases.

In our study; there were significant reduction in both total NK cells and mature NK cells in MDS-AML subgroup as compared to MDS subgroup that is not transformed. There is no previous report concerning this issue. However; previous reports detected reduction in mature NK but not in total NK cells in primary AML as compared to MDS groups. This finding pointing out for the role of total NK and mature NK cells in the evolution of AML on top of MDS. Sanchez-Correa et al., (2016) found that NK cells in AML patients showed diminished expression of several activating receptors that contribute to impaired NK cell function and, in consequence, to AML blast escape from NK cell immune surveillance. In addition; Costello et al., (2002) have done invitro experiment and found that there are impaired NK cell function and also cytokine production in AML. Also, in state of achievement complete remission the NK activity was improved (Fauriat et al., 2007). The antileukemic activity was attributed to memory like T cells which enhance interferon gamma production and cytotoxicity. This was proofed both in vitro studies on either leukemic cell lines or primary AML blasts (Romee et al., 2016). Furthermore; Human memory like NK cells xenografted into mice reduced AML burden in vivo and improved overall survival. Moreover; Gleason et al., (2014) reported that CD16XCD33 bispecific killer cell engager (BiKE) activates NK cells against primary MDS and MDSC CD33 targets and reverse immune suppression of NK cells and induced myeloid suppressor cells target cell lysis.

The total NK cell percentages as well as the mature NK cells were significantly reduced in MDS-AML group as compared to the remaining MDS group. These findings were not in agreement with that reported by Aggarwal et al., (2016) who found that there were significant differences in percentage of NK and mature NK cells in AML with poor prognosis and MDS patients’ group. This controversy could be explained according to Warilck and Miller (2011) statement who mentioned that some forms of MDS is due to immune dysregulation, while in others the abnormal immune function my represent only a small part. Furthermore; Stingaris et al., (2016) reported that the median number of activating KIRs was lower in MDs patients than normal controls and lower in patients with MDs-AML compared to de-novo AML patients. Moreover, they reported that lower expression of KIR in MDS patients independently predicted a higher risk conversion to AML. 

Alteration in the expression of cell-surface proteins is a common consequence of malignant transformation. Natural killer (NK) cells use an array of germline-encoded activating and inhibitory receptors that scan for altered protein-expression patterns, but tumor evasion of detection by the immune system is now recognized as one of the hallmarks of cancer. NK cells display rapid and potent immunity to metastasis or hematological cancers, and major efforts are now being undertaken to fully exploit NK cell anti-tumor properties in the clinic (Guillemet et al., 2018; Zhang et al., 2018).

The bone marrow soluble TNFα and IL-32 levels in all studied groups revealed that levels of both cytokines were significantly higher in AML-MDS group; followed by MDS group then the control group and the differences were statistically significant. Similar findings were reported by Marcondes et al., (2008) who stated that both TNFα and IL32 appears to deliver proapoptotic signals to KG1a cells which contributes to hematopoietic failure.

In the current study there were significant negative correlation between bone marrow sTNFα and sIL32 and the absolute neutrophil counts in the peripheral blood. These findings were consistent with that reported by Marcondes et al., (2008) and Warlick and Miller et al., (2011) who observed in their in vitro study that exposure of human marrow stroma cell lines HS5 and HS27a to TNFα increases the expression levels of IL-32 mRNA which in turn induces the production of TNF-α from the marrow stroma of patients with MDS. The increased IL-32 mRNA was 14- to 17-fold higher than healthy controls.

In conclusion, NK cells might have a role in AML evolution on the top of MDS. Likewise, the bone marrow soluble TNF-α; and IL-32 might have a role in MDS cytopenia’s. These finding may suppose novel therapeutic strategies for MDS. 
